# Depression and Associated Factors in Patients with Implantable Cardioverter-Defibrillators

**Published:** 2016-10-03

**Authors:** Maryam Moshkani Farahani, Mehrdad Taghipour, Mohsen Motalebi, Ramezan Bakhshian, Ehsan Shahverdi, Sovaid Taghipour, Soheyla Abdi, Ramzan Moradi

**Affiliations:** 1 *Atherosclerosis Research Center, Baqiyatallah University of Medical Sciences, Tehran, Iran.*; 2 *Nephrology and Urology Research Center, Baqiyatallah University of Medical Sciences, Tehran, Iran.*; 3 *Student Research Committee, Baqiyatallah University of Medical Sciences, Tehran, Iran.*; 4 *Islamic Azad University, Babol Branch, Babol, Iran*.; 5 *Baqiyatallah University of Medical Sciences, Tehran, Iran.*; 6 *Department of InfectiousDiseases, Mazandaran University of Medical Sciences, Sari, Iran.*

**Keywords:** *Depression*, *Defibrillators- implantable*, *Risk factors*

## Abstract

** Background:** Psychological problems such as depression constitute a dilemma that patients with the implantable cardioverter-defibrillator (ICD) usually encounter and may impact their quality of life. The purpose of this study was to evaluate the prevalence of depression among adults with the ICD and the relationship between depression and associated factors.

** Methods: **Totally, 115 individuals with the ICD participated in this cross-sectional study in Tehran, Iran, and depression and other related risk factors were evaluated in them. Two questionnaires, one for demographic characteristics and the Beck Depression Inventory, were applied for data acquisition. The data were analyzed, and the factors associated with depression in the patients with the ICD were assessed.

** Results: **The mean age of the study population was 59.85 ± 11.46 years. Males comprised 88 (76.5%) and females 27 (23.5%) of the patients. The multivariate analysis on the 115 patients revealed that frequency of ICD shocks (OR = 1.08, 95%CI: 1.02 - 1.10), male sex (OR = 2.28, 95%CI: 1.027 - 5.07), more hospital admissions (OR = 1.19, 95%CI: 1.11 - 1.25), smoking cessation (OR = 9.8, 95%CI: 4.48 - 20.07), presence of ICD shocks (OR = 4.5, 95%CI: 2.45 - 7.38), dyslipidemia (OR = 2.8, 95%CI: 1.22 - 4.95), and family history of depression (OR = 6.4, 95%CI: 3.0 - 13.46) were significantly and independently associated with the Beck score classifications.

**Conclusion: **These findings suggest that a poor psychosocial outcome in patients with the ICD may occur as a result of a variety of associated factors, most of which are predictable and preventable.

## Introduction

Cardiovascular diseases are known as the leading cause of death in industrialized nations and a significant etiologic factor for mortality in developed countries.^   1 ^ Based on studies conducted in Iran, cardiovascular diseases are culpable for the greatest burden of disease.^   2 ^ The World Health Organization (WHO) has reported an annual mortality of about 16.7 million for affected people. 

Although coronary artery diseases are the most common fatal cardiac diseases, arrhythmias, especially ventricular tachyarrhythmias, may lead to complications and prove life-threatening, if left untreated. Cardiopulmonary resuscitation can provide circulatory support, but the only effective way to terminate ventricular fibrillation is electrical defibrillation.^        3 ^ Patients who would benefit from the placement of the implantable cardioverter-defibrillator (ICD) are those who have a history of coronary artery diseases with a recent heart attack leading to heart failure, conditions that affect the heart muscle such as dilated cardiomyopathy, hypertrophic cardiomyopathy, and congenital heart defects that lead to abnormalities in heart rate such as long QT syndrome.^        4 ^

A number of studies have shown some serious psychological problems after the shock induced by the ICD and stated that these disturbances occur in about 13 to 38% of the patients as anxiety and 50% as depression. Younger patients, women, and those who receive ICD shocks are more prone to depression.^        5 ^ Various and sometimes conflicting theories are listed concerning these psychiatric and psychological problems. Some of these patients tend to be isolated and, as such, have their daily activities reduced due to a fear of an electrical discharge or shock, which can underlie depression. Depression is strongly associated with mortality in these patients. It seems that cognitive behavioral therapy and psychology training programs can reduce psychosocial deficits in these patients.^   6 ^ This suggests that attention to the psychological state of patients with the ICD is a therapeutic priority. 

Difficulties dealing with the complete management and treatment of the disease can originate from the shortcomings and contradictions in the findings of studies and available resources. In the present study, we aimed to overcome these key challenges and provide a context based on useful data for the medical community.

## Methods

From February 2013 to November 2014, the current study was conducted in Baqiyatallah Hospital (referral governmental hospital) and Jamaran Hospital (general hospital) in Tehran, Iran, to evaluate patients (≥ 18 y) with the ICD. The study was approved as a research project by the Ethics Committee of Baqiyatallah University of Medical Sciences. 

The inclusion criteria encompassed candidacy for ICD placement due to cardiac electrical disturbances, age ≥ 18 years, and the possibility of follow-up. Patients undergoing heart transplantation, patients with a history of severe psychiatric disorders such as psychotic disorders, bipolar disorders, major depressive disorder, and panic disorder and also patients with concomitant debilitating physical diseases such as multiple sclerosis, substance abuse, and history of an embedded ICD were excluded from the study.

The confirmation of the inclusion and exclusion criteria was based on clinical records and consultation with specialists in psychiatry and cardiology. The available sampling method was used to include all eligible patients in the study and complete the calculated sample size. The study sample size was determined considering a first-degree error of 5% and a study power of 80% and using the sample size calculation formula in a population with a determined Beck score variance.^17^

First, a list of patients eligible for inclusion in the study was extracted. These patients were thereafter invited for interviews, during which they filled out the questionnaires under the supervision of supervisors. Two questionnaires were applied for this study: a questionnaire for collecting demographic data and the Beck Depression Inventory (BDI) for assessing depression. A series of clinical information was extracted from the patients’ files in the archives during their hospital stay. All the patients signed an informed written consent before their inclusion in the study.

All ethical issues such as conflict of interest, misconduct, co-authorship, and double submission were considered carefully. Ethical permission for the study was obtained from the Ethics Committees of Baqiyatallah University of Medical Sciences and the participating hospitals. The ethical principles of autonomy, beneficence, non-maleficence, fidelity, and confidentiality were adhered to. 

All the continuous variables were tested for normal distribution before further statistical analyses with the Shapiro–Wilk test using STATA software, version 11.2. The distributional properties of the continuous data are expressed as means ± standard deviations. The categorical data are presented as frequencies and percentages. The differences between the Beck score and the classified qualitative variables were analyzed using the one-way analysis of variance, and the Kruskal–Wallis test or the Mann–Whitney U test was used for the continuous variables. The Spearman correlation test was used for the correlation between the variables with a non-normal distribution. An ordinal regression model was also employed to examine the association between the total Beck score and the other factors. Because the response variables were classified, we measured the associated factors with ordinal logistics regression. Also in order to achieve the final model, we utilized the backward method. A p value < 0.05 was considered statistically significant. For the statistical analyses, the statistical software SPSS version 18.0 for Windows (SPSS Inc., Chicago, IL) was used.

## Results

Of the 115 participants enrolled in this study at a mean age of 59.85 ± 11.46 years, 88 (76.5%) were male and 27 (23.5%) female. The mean time since ICD placement was 30.49 ± 24.02 months. The patients were asked about the duration of smoking and the number of cigarettes consumed per day (calculated as packs per year). The mean of the duration of cigarette smoking was 6.82 ± 13.53. Some patients were noted to have ICD-induced shocks repeated several times; the mean of shock frequencies was 2.38 ± 1.75 times. Another factor evaluated was the time interval between the last ICD shock and the study commencement, and the mean calculated time was 5.13 ± 12.04 months. Moreover, an important finding was a mean BDI score of about 12.96 ± 8.98.

Most of the patients were in the age range of between 50 and 59. The majority of the male patients (54 patients) were retired or disabled due to cardiovascular problems. A large percentage of the patients had irregular physical activity or none on a daily or weekly basis. The majority of the patients (31.3%) were found to be normal in terms of depression. However, depression was severe and more than severe in 7 patients, who needed therapeutic strategies. Significant differences were seen between sex and occupation (p value = 0.000), education (p value = 0.016), marital status (p value < 0.001), and smoking (p value = 0.002). 

The total Beck score and the quantitative and qualitative variables were compared. The results showed that smoking duration, frequency of ICD shocks, and the time interval between the last ICD shock and the study commencement were significantly correlated with the mean Beck score. Additionally, the univariate comparison between this index (Beck score) and some other factors such as family history of depression and age classification revealed that there were some statistically significant differences in some cases (Table 1). An ordinal logistic regression model was performed in order to assess the relationship between the Beck classification and smoking, sex, marital status, hospital admissions, and some other factors (Table 2). The relationships between the variables and the Beck score were all significant. Smoking cessation (OR = 9.82, 95%CI: 4.48 - 20.07), male sex (OR = 2.28, 95%CI: 1.027 - 5.07), higher shock frequencies (OR = 1.08, 95%CI: 1.02 - 1.10), more hospital admissions (OR = 1.19, 95%CI: 1.11 - 1.25), presence of ICD shocks (OR = 4.51, 95%CI: 2.45 - 7.38), dyslipidemia (OR = 2.8, 95%CI: 1.22 - 4.95), and family history of depression (OR = 6.46, 95%CI: 3.03 - 13.46) were more likely to be associated with higher grades of depression, whereas longer durations of smoking, concomitant disease, and longer interval between ICD placement and study commencement were less likely to be correlated with higher grades of depression.

**Table 1:** Univariate comparisons between demographic and clinical characteristics and the mean Beck score of the patients with the ICD*

Variable	Total number	Mean Beck score	P value
Age (y)			0.169
< 40	3 (2.6)	6.33±3.78	
40-49	16 (13.9)	18.37±13.87	
50-59	42 (36.5)	12.00±8.00	
60-69	26 (22.6)	13.92±8.37	
≥ 70	28 (24.3)	11.14±6.54	
Occupation			0.313
Jobless	31 (27.0)	13.93±9.89	
Government employee	13 (11.3)	14.38±11.34	
Self-employed	17 (14.8)	9.82±8.54	
Retired	54 (47.0)	13.05±7.92	
Education			0.135
Diploma	41 (35.7)	12.97±9.18	
Associated degree	8 (7.0)	17.12±13.50	
Bachelor’s degree	18 (15.7)	8.83±4.84	
Master’s degree	8 (7.0)	9.00±4.20	
Other	40 (34.8)	14.77±9.23	
Exercise			0.119
None	49 (42.6)	14.53±8.89	
Irregular	34 (29.6)	12.44±9.55	
Regular	32 (27.8)	11.12±8.34	
Marital status			0.613
Single	0	0	
Married	107 (93)	12.79±9.03	
Divorced	4 (3.5)	14.25±4.71	
Widow	4 (3.5)	16.25±11.98	
Season			0.110
Spring	26 (22.6)	13.50±9.39	
Summer	37 (32.2)	14.56±8.53	
Autumn	19 (16.5)	9.57±7.45	
Winter	33 (28.7)	12.69±9.76	
Number of Children			0.111
< 3	72 (62.6)	12.62±9.84	
≥ 3	43 (37.4)	13.53±7.40	
Cardiac risk factors			0.011
HTN	30 (26.1)	14.43±8.15	
DM	24 (20.9)	17.54±9.83	
DLP	22 (19.1)	17.09±10.08	
Smoking	41 (35.7)	15.85±10.40	
Shock			≤ 0.001
Presence	47 (40.9)	16.14±10.09	
Lack	68 (59.1)	10.76±7.44	
History of hospitalization			0.214
Yes	87 (75.7)	13.56±9.20	
No	28 (24.3)	11.10±8.13	
FH of depression			0.833
Positive	16 (13.9)	11.93±8.26	
Negative	99 (86.1)	13.13±9.12	
Shock frequency	47 (40.86)	12.96±8.98	0.00
Duration of hospitalization	87 (75.7)	12.96±8.98	0.00

HTN, Hypertension; DM, Diabetes mellitus; DLP, Dyslipidemia

* Data are presented as n (%) or mean±SD.

**Table 2:** Final model for predictive factors of depression in patients with ICD by ordinal logistic regression

Factors	OR	CI 95%	P value
Duration of ICD replacement until study	0.97	0.97 - 0.99	< 0.001
Shock frequency	1.08	1.02 - 1.10	0.003
Admission time	1.19	1.11 - 1.25	0.00
Gender			
Male	2.28	1.027 - 5.07	0.043
Female	Base	-	
Marital status			
Single	0.15	0.04 - 0.67	0.008
Married	0.73	0.12 - 3.66	0.662
Divorced	Base	-	
Smoking			
Smoker	9.82	4.48 - 20.07	< 0.001
Non-smoker	Base	-	
Presence of ICD shock			
Presence of shock	4.56	2.45 - 7.38	< 0.001
Lack of ICD shock	Base	-	
Dyslipidemia history			
Positive	2.81	1.22 - 4.95	0.001
Negative	Base	-	
Presence of other diseases			
Presence of other diseases	0.12	0.08 - 0.24	< 0.001
No other diseases	Base	-	
Family history of depression			
Positive	6.40	3.06 - 13.46	< 0.001
Negative	Base	-	

## Discussion

Based upon the literature, there are few prospective studies evaluating depression over time in ICD recipients. The existing records show confusing and often contradictory data regarding the exact statistics of psychological disturbances associated with ICD placement. As it can be expected that the number of patients with the ICD will increase, the rate of psychosocial problems in these patients will also grow.^   7 ^ The current paper aimed to identify the association between depression and receiving ICD shocks and the existence of other factors. Our results demonstrated that the frequency of depression was about 17% accumulatively in terms of the BDI classifications; this finding is similar to those reported by some recent studies stating a range between 5% and 38%. ^8^^-^^10^ 

Some studies have investigated whether smoking confounds, mediates, or moderates the relationship between depression and cardiac diseases; they have, however, merely reported limited evidence of such relations. ^11^^, ^^12^  Our results are in contrast with their findings insofar as we found that in the patients with more attempts at smoking cessation, high grades of depression were more probable. Further clarification of this issue requires a comprehensive model of the mechanisms for the effect of smoking on cardiac diseases.

Gender seems to be a key factor in depression among ICD recipients.^   13 ^ This hypothesis may have originated from the possible role of genetic and environmental factors in depression. The evidence suggests that females are more prone to depression, while we found that the male sex was more susceptible to depression. ^14^^, ^^15^  The difference in the results may be explained by our small sample size and low number of female patients. 

Moreover, our regression analysis demonstrated that more hospital admissions, dyslipidemia, and family history of depression were more strongly correlated with a higher possibility of having depression in our patients with the ICD. 

In the current study, we considered the time of the last ICD shock. We included patients who had had received early or late ICD shocks or shocks at both stages because we believe that the time of ICD shock delivery affects the quality of life. Our results in this regard are very close to those reported previously. 

Tsuyoshi Suzuki et al.^        16 ^ showed that patients with depression had experienced more ICD shocks during the 2-year follow-up than did those without depression. Likewise, Kamphuis et al.^17^ and Schron et al.^18^ reported that the occurrence of defibrillator shocks was independently associated with a significant reduction in mental well-being in patients receiving the ICD.  These findings mean that depression is associated not only with experiencing ICD shocks, but also with increasing numbers of shocks. In our study, the number of shocks each person experienced was significantly associated with a higher possibility of developing high levels of depression.

The novel aspect of the present study is that we assessed the influence of various factors on depression with a view to addressing the existing gap in knowledge regarding psychiatric and psychological problems in patients with the ICD. Various basic mechanisms have been proposed to be responsible for depression in this patient population so far in the literature; they include the role of inflammation, endothelial dysfunction, increased platelet activity and aggregation, neurohormonal and autonomic nervous system dysfunction, effects of the brain-derived neurotrophic factor (BDNF), and related behavioral factors.^19^^-^^27^

The results of the current study may have some practical implications. The relatively low rate of depression denotes that the majority of our patients had adjusted well with their device. Accordingly, clinicians seeking to better detect the possible presence of complications such as psychiatric and psychological problems should devote special attention to all ICD recipients who experience arrhythmias and not only to those who receive shocks. 

This study has some limitations. One limitation of this study is its low sample size, which may explain the nonsignificant differences in some cases. A study with equal numbers of men and women is recommended to provide accurate data on factors in both genders. In addition, the single use of the BDI test was another limitation of this study. Using other simultaneous tests along with the BDI and also clinical psychiatric interviews seems to be necessary. In our evaluation of the association between total depression levels (as defined in the body of the paper) and other factors, we were only able to analyze the overall statistics. Nevertheless, to determine the exact relationship between factors associated with depression and subclasses of depression, we need to perform another regression model. 

The identification of the high prevalence of depression among our patients with the ICD supports previous recommendations, denoting that these patients need psychological care and education by professional teams in order that their dependence on health care services can be reduced. 

## Conclusion

From this study, it can be concluded that psychiatric consultation should be the first recommended option for patients with the ICD, especially individuals with concomitant risk factors for depression. In such patients, depression can be treated via different methods. ICD-specific education, including how the device functions and what to do when a shock occurs, is highly recommended. These educational strategies, in combination with cognitive behavioral therapy (CBT) strategies, can enhance the mood and quality of life of ICD recipients. Be that as it may, determining the optimal treatment for decreasing the possibility of psychological complications continues to be a problem facing future studies.

## Notes:

***This paper should be cited as:*** Moshkani Farahani M, Taghipour M, Motalebi M, Bakhshian R, Shahverdi E, Taghipour S, Abdi S, Moradi R. Depression and Associated Factors in Patients with Implantable Cardioverter-Defibrillators. J Teh Univ Heart Ctr 2016;11(4):168-173
